# Infection with diverse immune-modulating poxviruses elicits different compositional shifts in the mouse gut microbiome

**DOI:** 10.1371/journal.pone.0173697

**Published:** 2017-03-10

**Authors:** Daniel Aguirre de Cárcer, Bruno Hernáez, Alberto Rastrojo, Antonio Alcamí

**Affiliations:** Centro de Biología Molecular Severo Ochoa, Consejo Superior de Investigaciones Científicas (CSIC)–Universidad Autónoma de Madrid (UAM), Madrid, Spain; Instituto de Agroquimica y Tecnologia de Alimentos, SPAIN

## Abstract

It is often not possible to demonstrate causality within the context of gut microbiota dysbiosis-linked diseases. Thus, we need a better understanding of the mechanisms whereby an altered host immunophysiology shapes its resident microbiota. In this regard, immune-modulating poxvirus strains and mutants could differentially alter gut mucosal immunity in the context of a natural immune response, providing a controlled natural *in vivo* setting to deepen our understanding of the immune determinants of microbiome composition. This study represents a proof-of-concept that the use of an existing collection of different immune-modulating poxviruses may represent an innovative tool in gut microbiome research. To this end, 16S rRNA amplicon sequencing and *RNAseq* transcriptome profiling were employed as proxies for microbiota composition and gut immunophysiological status in the analysis of caecal samples from control mice and mice infected with various poxvirus types. Our results show that different poxvirus species and mutants elicit different shifts in the mice mucosa-associated microbiota and, in some instances, significant concomitant shifts in gut transcriptome profiles, thus providing an initial validation to the proposed model.

## Introduction

The gut microbiome is an influential factor underpinning various health challenges faced by societies today. A myriad of research articles have linked shifts in gut microbial community structure (dysbiosis) to numerous human diseases (reviewed by Pflughoeft & Versalovic [1]). Unfortunately, it has been often not possible to unravel whether the dysbiosis was the causative agent (or coadjuvant) of host disease or rather the expected consequence of the altered host immunophysiology. Causality within the context of dysbiosis-linked diseases has been demonstrated in some instances using microbial transplantation experiments. For example, a colitis phenotype was transferable from Tbx21^−/−^/Rag2^−/−^ mice to wild-type mice by transfer of the implicated microbiota [2]. However, we need a better understanding of the mechanisms whereby an altered host immunophysiology shapes microbiota composition.

Poxviruses have evolved mechanisms to control key components of the immune system. *Vaccinia* virus (VACV, the smallpox vaccine) and *Ectromelia* virus (ECTV, the causative agent of mousepox) encode soluble proteins that bind interferon, cytokines or chemokines to modulate immunity [3,4]. The infection of mice with VACV and ECTV mutants deficient in immune modulatory proteins represents a unique model system to dissect immune pathways, and has produced important advances in the fields of virology and immunology [5,6,7]. VACV and ECTV mutants lacking the type I IFN binding protein cannot control the host IFN response and are highly attenuated [8,9]. Poxviruses encode four tumour necrosis factor (TNF) receptors that efficiently block responses triggered by TNF [10]. Two of the viral TNF receptors have an additional domain that binds chemokines and inhibits chemokine-induced cell migration [11].

Recently, Deriu et al. [12] have shown in mice that influenza pulmonary infection alters the gut microbiota through a mechanism dependent on type I IFN. The present study represents a proof-of-concept that the use of immune-modulating poxviruses may represent a valuable tool in gut microbiome research. These viruses could differentially alter gut mucosal immunity in the context of a natural immune response, and hence, through the joint monitoring of gut mucosal immunity and microbiota composition, help increase our understanding of the immune determinants of microbiome composition.

In this study we employed 16S rRNA amplicon sequencing and transcriptome profiling as proxies for microbiota composition and gut immunophysiological status, respectively. This approach was employed to analyze caecal samples from mice uninfected or infected with various poxvirus species and mutants. Our expectation was that the different viruses would elicit different shifts in both gut microbiota composition and host transcriptome patterns, hence providing an initial validation to our hypothesis.

## Materials and methods

All samples analyzed in this study originate from mice sacrificed as part of a larger-scale effort to understand poxvirus modulation of immune pathways. As such, sample size was determined by the needs of the mentioned study. All animal experiments were performed in compliance with national and international regulations and were approved by the Ethical Review Board of Centro de Biología Molecular Severo Ochoa and Consejo Superior de Investigaciones Científicas under reference CEEA-CBMSO-16/080 and project number SAF2012-38957. We employed wild-type ECTV (Ewt, strain Naval) [13], its ECTVΔCrmD knockout mutant deficient in the secreted TNF receptor CrmD (EdCrmD), wild-type VACV (Vwt, strain Western Reserve), and its VACV deletion mutant lacking expression of the interferon type I binding protein B18 (VdB18) [9]. CrmD binds TNF and a few chemokines, is a major virulence factor [11], and exhibits a potent anti-inflammatory activity (A. Alejo, M. B. Ruiz-Argüello, S. M. Pontejo, M. M. Fernández de Marco, M. Saraiva and A. Alcami, personal communication). The CrmD deficient mutant has been shown to activate host´s TNF signaling, and the B18 deficient mutant is unable to block the IFN-based host response. Both mutant viruses (EdCrmD and VdB18) exhibit an attenuated phenotype in infected mice when compared to the corresponding parental viruses (A. Alejo, M. B. Ruiz-Argüello, S. M. Pontejo, M. M. Fernández de Marco, M. Saraiva and A. Alcami, personal communication)[9]. Viruses were purified by centrifugation through a sucrose cushion prior to animal inoculation.

Female Balb/c (4–6 weeks old) from Charles River Laboratories were anesthetized with isofluorane and infected or mock-infected with 10 μl of indicated virus inoculums. In the case of ECTV, animals were infected subcutaneously with 10^3^ plaque-forming units (pfu) by footpad inoculation. For VACV infections, 10^4^ pfu were intranasally inoculated. In the case of mock-infections, animals were inoculated intranasally and subcutaneously with 10 μ l of PBS-0.1% BSA. Mice were housed in ventilated racks under biological safety level 3 containment facilities until they were sacrificed. Mice were euthanized by CO_2_ asphyxiation. Investigators allocating mice to experimental groups were unaware of the final groups, hence mice were randomly grouped to receive viruses.

Caeca (n = 5) were aseptically removed from infected and mock-infected animals. Initially, samples were obtained at day 2 post infection (dpi), representing early infection, as well as at a later infection stage (5 and 7 dpi for VACV and ECTV, respectively. These samples represent the first batch). Once the most appropriate sampling time was established (see below), another batch of samples (n = 5 mice per experimental group) at 2 dpi was independently obtained (batch 2). Mice ceca were quickly immersed in *RNAlater* preserving solution (Qiagen) and stored at 4°C for one day. After that, samples were stored at -20°C until processed. During sample processing, the investigators were unaware of the sample groupings. Caeca were cut longitudinally, with one section kept for DNA extraction and microbial community profiling, and the other employed for host transcriptome profiling. Before subsequent processing, luminal contents were washed from the samples; caecal moieties for transcriptomic profiling were briefly vortexed in clean *RNAlater* solution, while those intended for community profiling were briefly washed by vortexing in sterile PBS then frozen.

For microbiome profiling, community DNA was extracted using PowerSoil DNA isolation kit (MoBio, Carlsbad, CA) according to the manufacturer’s instructions, and then measured using a Nanodrop ND-1000 spectrophotometer (Nanodrop, Wilmington, DE). Bacterial 16S rRNA marker genes were amplified from the resulting DNA samples following a nested strategy, featuring a bacteria-specific PCR following by a short-cycle PCR reaction to add barcodes, modified from that described by Lunbderg et al [14]. Briefly, the first primer pair was formed by group specific oligonucleotides targeting positions 341 and 805 of the 16S rRNA gene [15], followed (3’-5’) by a 2 bp linker, 1–4 Ns for frame shifting to increase Illumina sequencing efficiency, and shorter linker sequences (CS1, 5’-ACACTGACGACATGGTTCTACA-3’; CS2 5’- TACGGTAGCAGAGACTTGGTCT-3’). The second primer pair consisted (5’-3’) of Illumina’s P5 sequence followed by CS1, and Illumina’s P7 followed by 9 bp barcodes and CS2. Initial bacteria-specific PCRs were carried out using 20 ng of template DNA, 12.5 μ l of 2× iProof high-fidelity DNA polymerase mix (Bio-Rad, Hercules, CA) and 5 pmol of each primer. PCR reaction conditions included an initial denaturation step of 2 min at 98°C, followed by 25 cycles of 10s at 98°C, 30s at 55°C, 30s at 72°C, and a final elongation step of 5 min at 72°C. Aliquots of the resulting products were monitored by gel electrophoresis before primer removal by *ExoI* reaction [16]. Final barcoding PCRs were performed with 6.5 μ l of clean PCR products from the group-specific PCR, 5 pmol of the general and barcoded primers and 12.5 μ l of 2× iProof high-fidelity DNA polymerase mix, following the same reaction conditions as before but including only ten reaction cycles. The size and overall quality of the resulting products was again monitored by agarose electrophoresis, and a SequalPrep Normalization Plate (ThermoFisher) was employed to normalize amplicon concentrations before pooling. The pooled amplicon library was gel extracted using the QIAquick Gel Extraction Kit (QIAGEN), and sequenced using an Illumina MiSeq device (2 x 300 bp). Unless otherwise stated, QIIME [17] scripts were employed during sequence processing and analysis. After initial quality filtering, paired reads were merged, and bacteria-specific primer sequences clipped using in-house scripts. Then, chimeric sequences were identified (*usearch61*) and later removed. Subsequently, sequences were clustered *de novo* into OTUs (*usearch61*) at 0.97 distance. The OTU representative sequences were aligned to the Greengenes reference database, and taxonomically assigned. OTUs with representative sequences failing to align were also removed from the resulting OTU table before the final normalization step by randomly subsampling to a common depth (7 550 sequences per sample).

6 samples per experimental group were employed for transcriptome profiling. Total RNA was isolated using Reliaprep RNA tissue miniprep system (Promega) according to the manufacturer’s instructions. RNA samples were assessed for quantity on a spectrophotometer (NanoDrop ND-1000; Thermo Scientific) and for integrity in an Agilent 2100 Bioanalyzer (Agilent Technologies). Afterwards, a standard polyA selection was carried out using the Poly(A)mRNA Magnetic Isolation Module (New England Biolabs) according to the manufacturer’s instructions. Then, sequencing libraries were prepared using the Ultra Directional RNA Library Prep Kit (New England Biolabs). Finally, samples were sequenced using an Illumina NextSeq device (1 x 75 bp), yielding 35 867 651±6 364 958 sequences per sample (range between 22 395 015 and 58 581 379). Transciptome profiling was carried out by mapping the reads to the mouse transcriptome using *tophat2* [18] and results processed by *HTSeq* [19] to obtain gene counts, using ‘no-novel-juncs’ and ‘intersection_nonempty’ parameters, respectively. Profiles were then processed using the *DESeq2* package [20] in R using the *varianceStabilizingTransformation* function.

All subsequent statistical and analytical procedures were carried out in *R* (R Core Team 2013), mainly employing functions within package *ade4* [21]. Exploration of microbial community profiles was carried-out through principal component analysis and double principal coordinates analysis (DPCoA). The latter is an ordination method that takes into account phylogenetic (genetic distance) relatedness between OTUs when explaining variation in the data, hence quantifying community dissimilarity based on phylogenetic relatedness. The statistical significance of *a priori* community groupings was tested by analysis with respect to instrumental variables and constrained double principal coordinates analysis (cDPCoA) [22], in both cases using experimental batch as cofactor, followed by Monte Carlo permutation tests (within group analysis using experimental batch as constraint, followed by a between class analysis using experimental group as constraint). Statistical differences between major bacterial group abundances were assessed using a Student’s t-test. Finally, statistical significance of *a priori* community groupings for the transcriptome profiles were also undertaken using analysis with respect to instrumental variables followed by Monte Carlo permutation tests.

## Results and discussion

**Fig 1:**
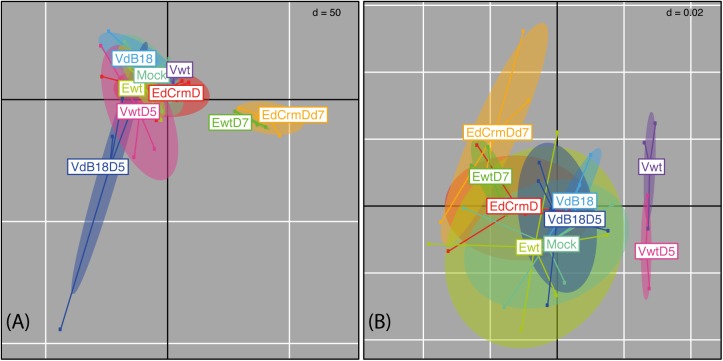
Exploration of trends in mucosa-associated caecal microbiome composition. Figures depict results from a principal components analysis (A) and Double Principal Coordinates Analyses (DPCoA) (B) based on 16S marker gene profiles. The analyses ordinate experimental groups based solely on the per-group relative abundances of OTUs (A), or based on the phylogenetic distances among OTUs and per- group relative abundances of OTUs (B). Sample points and experimental group labels are jointly colored, and ellipses describe the experimental groups’ collective variances. Early infection sample groups; Mock, Vwt, Ewt, VdB18, EdCrmD. Late infection sample groups; VwtD5, VdB18D5, EwtD7, EdCrmDd7.

The initial exploration of bacterial community profiles evidenced strong compositional shifts associated with late sampling times (Fig 1). This may reflect diverse signs of illness and pathology in different organs at late stages of infection. In the case of ECTV, subcutaneous infection of susceptible mice results in extensive necrosis and damage in spleen and liver after multiplication in these organs by 4 dpi, and also small intestinal mucosal erosions and engorged guts are frequently detected among other pathological changes at late stages [5,23]. VACV also causes a severe disease in susceptible mice after intranasal infection, with detectable weight loss and signs of illness by 5 dpi, associated with high virus titers in lungs and brain (9). Hence, to avoid potential confounding factors, we focused on early time points (2 dpi) when we hypothesized that the initial virus replication might have affected mucosal immunity in the gut and not yet triggered strong confounding factors (e.g. fever, loss of appetite, inflammation processes, modified liver function).

**Fig 2:**
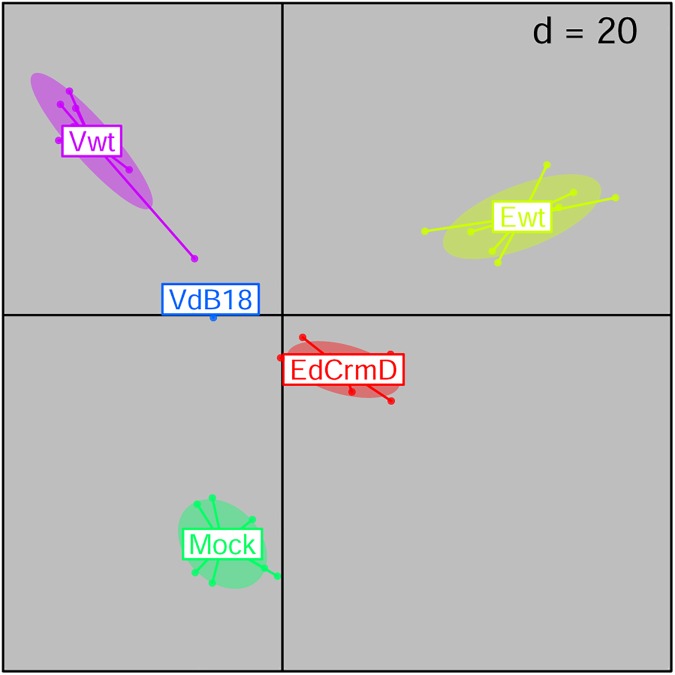
Exploration of trends in microbiome composition during early infection. The figure depicts results from an analysis with respect to instrumental variables applied to a principal components analysis based on 16S marker gene profiles (within group analysis using experimental batch as constraint, followed by a between class analysis using experimental group as constraint). The analysis ordinates samples (VdB18; n = 7. Ewt, Vwt, EdCrmD; n = 10) based on the similarity between their relative abundances of OTUs. Sample points and experimental group labels are jointly colored, and ellipses describe the experimental groups’ collective variances.

The analysis of bacterial community profiles at 2 dpi revealed significant differences associated with the experimental groupings (Fig 2. BCA; p<0.001), not only based on OTUs relative abundances but also when taking into account the phylogenetic relatedness among OTUs (cDPCoA; p<0.05). Such analysis also showed a pattern suggesting that both viral mutant profile groups represent a midpoint between the effect caused by infection with the wild-type viruses and the mock-infected community (Fig 2). Interestingly, the results also indicated that infection with Ewt and Vwt shift community structure towards different compositional states. Subsequent individual pairwise comparisons among experimental groups (Table 1) established that each treatment group presented a distinct (p<0.05) pattern of community composition. Focusing on the wild-type and mock experimental groups, we observed significant differences in the relative abundance of some major bacterial taxa (Table 2); the percentage of sequences affiliated to Deferribacteres were two-fold lower in Vwt samples (p<0.05), and those assigned as Proteobacteria were over three-fold lower in Ewt samples (p<0.05), when compared to the mock-infected group. However, overall Shannon diversity values were not significantly affected by these infections (8.3±3, 8.2±6 and 8.0±4 for mock, Ewt and Vwt infections respectively).

**Table 1:** Differences between community compositions. Results represent p-values of Monte Carlo permutation tests assessing the significance of experimental groups after controlling for possible batch effects.

	BCA^1^
**Mock** vs. **Vwt**	0.002*
**Mock** vs. **Ewt**	0.010*
**Mock** vs. **VdB18**	0.035*
**Mock** vs. **EdCrmD**	0.036*
**Vwt** vs. **VdB18**	0.002**
**Ewt** vs. **EdCrmD**	0.032*
**Vwt** vs. **Ewt**	0.002**

^**1**^BCA [Between class analysis]. **Sign**: ** (p<0.01), * (p<0.05). Adjusted for multiple testing (FDR).

**Table 2:** Relative abundance (%) of major bacterial groups for Mock, ECTV and VACV infections. ± represent SD.

	Ewt	Mock	Vwt
Firmicutes	51.2 ± 7.5	53.5 ± 6.6	55.4 ± 6.5
Bacteroidetes	27.8 ± 6.8	23.7 ± 3.6	28.6 ± 8.6
Deferribacteres	9. 2 ± 5.1	8.3 ± 4.6	4.1 ± 5.0
Proteobacteria	1.1 ± 0.6	4.1 ± 4.2	3.2 ± 3.3
Actinobacteria	1.6 ± 0.7	1.2 ± 0.5	0.8 ± 0.5
Tenericutes	0.8 ± 0.9	1.2 ± 1.8	1.4 ± 1.7

**Fig 3:**
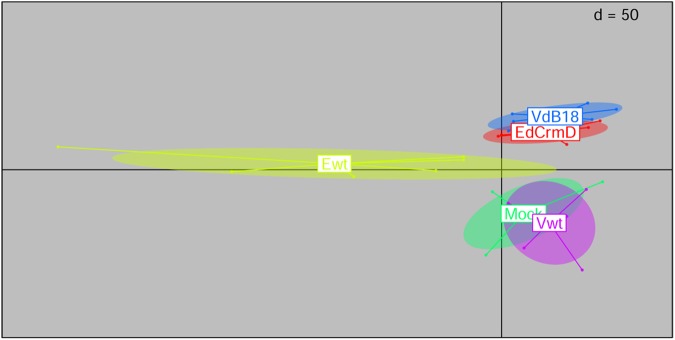
Exploration of trends in host caecal transcriptomic profiles during early infection. The figure depicts results from an analysis with respect to instrumental variables applied to a principal components analysis based on transcriptome profiles (within group analysis using experimental batch as constraint, followed by a between class analysis using experimental group as constraint). The analysis ordinates samples (n = 6) based on the similarity between their trancriptome profiles. Sample points and experimental group labels are jointly colored, and ellipses describe the experimental groups’ collective variances.

Host caecal transcriptomic profiles were used as proxies for gut immunophysiological state. Overall, the RNA integrity values (RIN) obtained were relatively low (4.5 ± 1), likely due to the fact that the gut is a very delicate tissue when it comes to mRNA extraction [24]. In this regard, partially degraded mRNA could impact downstream analyses. However, as RIN values were not correlated with any experimental grouping, the effect would mainly produce increased experimental noise which could hinder the resolution of putative differential expression patterns among experimental groups. Nevertheless, transcriptome profiling analysis uncovered at least significant differences when comparing Ewt samples to other experimental groups (Fig 3). Similarly, ensuing pairwise comparisons detected significant differences (p<0.05) when comparing Ewt with all other experimental groups. Subsequent comparison of Ewt vs. Mock-infected transcriptome profiles using pathway analysis tools (IPA, QIAGEN) revealed an inflammatory state related to various immune processes (S1 Fig, S1 Mat).

The present proof-of-concept study shows that the different infections with the immune-modulating viruses elicit different changes in gut microbiome community structure and, in most cases, also overall phylogenetic composition. In addition, and despite the probable increased noise derived from the low RIN values associated with the mRNA samples, at least the infection with ECTV produced concomitant shifts in gut transcriptome profiles. Hence, these results provide an initial validation to the idea that a collection of viruses with different ability to modulate the immune response may represent a valuable tool in gut microbiome research. Nevertheless, further research is necessary to more adequately determine the effect of infection in gut transcriptome profiles.

## Conclusion

The use of germ-free mice-based models has increased our understanding of the immunophysiological effects elicited by bacteria on the host. Unfortunately, the other side of the equation; the effect that changes in host immunophysiology may have on the resident microbiota remains understudied, mainly due to the lack of proper experimental models. In this respect, Knock-out mice have been the main experimental models employed, also serving as models of diseased states (e.g. T-bet-/-xRAG2-/- mice and Ulcerative Colitis [25]. While their use provides the theoretical opportunity to dissect the host genetic component of the host-microbiome interacting system, it represents a rather brute force approach as these deficient strains present dysbiotic states *per se*. which hinders the use of proper negative controls. The approach we propose could be employed alone or jointly with germ-free, knock-out, and (or) gnotobiotic approaches to reproducibly and effectively advance the science of host-microbial mutualism. For instance, our approach should allow the detection of links between the activity of different modules of gut immunity and the abundance or activity of particular groups of bacteria.

## Supporting information

S1 Fig Top 20 enriched IPA pathways in caecum after Ectromelia virus infection. Significant and differentially expressed genes from ECTV infected samples compared to control uninfected were used in a pathway enrichment analysis using IPA software (Ingenuity). Top 20 most enriched pathways and their corresponding p-values are shown. Lines connect overlapping pathways sharing at least 5 genes.
(PDF)

S1 Mat Results from the Ingenuity Pathway Analysis. (XLS)
