# Supplementary material for: Infection with diverse immune-modulating poxviruses elicits different compositional shifts in the mouse gut microbiome
Source: PLoS One. 2017 Mar 10;12(3):e0173697. doi: 10.1371/journal.pone.0173697 (PMC5345840; doi:10.1371/journal.pone.0173697)
Supplement: S1 Fig — Significant and differentially expressed genes from ECTV infected samples compared to control uninfected were used in a pathway enrichment analysis using IPA software (Ingenuity). Top 20 most enriched pathways and their corresponding p-values are shown. Lines connect overlapping pathways sharing at least 5 genes. (PDF) [file pone.0173697.s001.pdf]

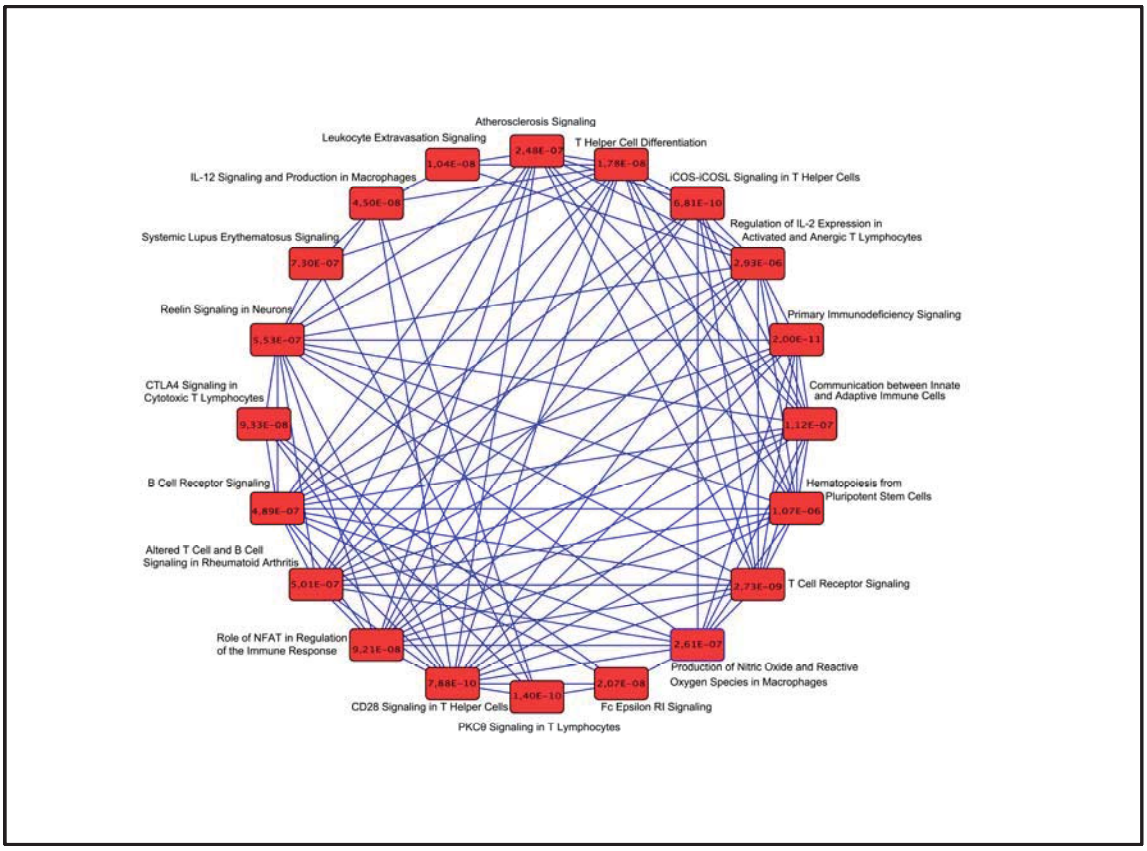

Supplementary Figure 1. **Top 20 enriched IPA pathways in caecum after Ectromelia virus infection.** Significant and differentially expressed genes from ECTV infected samples compared to control uninfected were used in a pathway enrichment analysis using IPA software (Ingenuity). Top 20 most enriched pathways and their corresponding p-values are shown. Lines connect overlapping pathways sharing at least 5 genes.
